# Supplementary material for: Evaluating tramadol utilization and patterns by county-level social determinants of health characteristics from 2015 to 2022
Source: medRxiv. 2025 Nov 6:2025.11.03.25339433. Preprint. [Version 1] doi: 10.1101/2025.11.03.25339433 (PMC12637732; doi:10.1101/2025.11.03.25339433)
Supplement: 1 [file NIHPP2025.11.03.25339433V1-supplement-1.pdf]

medRxiv preprint doi: https://doi.org/10.1101/2025.11.03.25339433; this version posted November 6, 2025. The copyright holder for this preprint (which was not certified by peer review) is the author/funder, who has granted medRxiv a license to display the preprint in perpetuity.
It is made available under a CC-BY 4.0 International license .

Supplementary Information

Figure S1:

(A) Top 3 States with the Highest Proportion of Tramadol Prescriptions from Prescribers Outside West Virginia
(B) Top 3 Counties with the Highest Proportion of Tramadol Prescriptions from Prescribers Outside West Virginia
(C) Top 3 States with the Highest Proportion of Tramadol Prescriptions Filled in Pharmacies Outside West Virginia
(D) Top 3 Counties with the Highest Proportion of Tramadol Prescriptions Filled in Pharmacies Outside West Virginia
The proportions were calculated for Figure 4A & 4B using the total prescriptions from each state or county as a percentage of the total number of tramadol prescriptions written by an out-of-state prescriber in each year (2015 N = 50062, 2016 N = 45576, 2017 N = 38719, 2018 N =30371, 2019 N = 25219, 2020 N = 23926, 2021 N = 24099, 2022 N = 23852).
The proportions were calculated for Figure 4C & 4D using the total prescriptions from each state or county as a percentage of the total number of tramadol prescriptions filled in an out-of-state pharmacy in each year *(2015 N = 8888, 2016 N = 8775, 2017 N = 8898, 2018 N = 8102, 2019 N = 9218, 2020 N = 11429, 2021 N = 12691, 2022 N = 13310*)

Table S1a: Percentage of Tramadol Prescriptions Issued by an Out-of-State Prescriber

Table S1b: Percentage of Tramadol Prescriptions Filled in an Out-of-State Pharmacy

Figure S2: Tramadol Co-Prescription by Number & Type Per Year

The percentage was calculated using the number of patients who had multiple prescriptions including tramadol in the same month of a particular year and the total number of patients prescribed tramadol for that year (2015 N = 127089, 2016 N = 120078, 2017 N = 105148, 2018 N = 91044, 2019 N = 81392, 2020 N = 71897, 2021 N = 69314, 2022 N = 66953)

Table S2: Percentage of Patients with Tramadol Co-Prescription by Number and Type of Prescription Medicines
